# Transcutaneous Intraluminal Impedance Measurement for Minimally Invasive Monitoring of Gastric Motility: Validation in Acute Canine Models

**DOI:** 10.1155/2014/691532

**Published:** 2014-12-09

**Authors:** Michael D. Poscente, Gang Wang, Dobromir Filip, Polya Ninova, Gregory Muench, Orly Yadid-Pecht, Martin P. Mintchev, Christopher N. Andrews

**Affiliations:** ^1^Centre for Bioengineering and Research, University of Calgary, Engineering Complex, 2500 University Drive NW, Calgary, AB, Canada T2N 1N4; ^2^Department of Electrical and Computer Engineering, University of Calgary, Engineering Complex, 2500 University Drive NW, Calgary, AB, Canada T2N 1N4; ^3^Division of Pediatrics, Faculty of Medicine, University of Calgary, Calgary, AB, Canada T2N 1N4; ^4^Faculty of Veterinary Medicine, University of Calgary, Calgary, AB, Canada T2N 1N4; ^5^Department of Surgery, Faculty of Medicine, University of Alberta, Edmonton, AB, Canada T6G 2B7; ^6^Division of Gastroenterology, Faculty of Medicine, University of Calgary, Calgary, AB, Canada T2N 1N4

## Abstract

Transcutaneous intraluminal impedance measurement (TIIM) is a new method to cutaneously measure gastric contractions by assessing the attenuation dynamics of a small oscillating voltage emitted by a battery-powered ingestible capsule retained in the stomach. In the present study, we investigated whether TIIM can reliably assess gastric motility in acute canine models. *Methods*. Eight mongrel dogs were randomly divided into 2 groups: half received an active TIIM pill and half received an identically sized sham capsule. After 24-hour fasting and transoral administration of the pill (active or sham), two force transducers (FT) were sutured onto the antral serosa at laparotomy. After closure, three standard cutaneous electrodes were placed on the abdomen, registering the transluminally emitted voltage. Thirty-minute baseline recordings were followed by pharmacological induction of gastric contractions using neostigmine IV and another 30-minute recording. Normalized one-minute baseline and post-neostigmine gastric motility indices (GMIs) were calculated and Pearson correlation coefficients (PCCs) between cutaneous and FT GMIs were obtained. Statistically significant GMI PCCs were seen in both baseline and post-neostigmine states. There were no significant GMI PCCs in the sham capsule test. Further chronic animal studies of this novel long-term gastric motility measurement technique are needed before testing it on humans.

## 1. Introduction

Distal postprandial gastric motility involves contractions that mechanically crush ingested food and mix it with secretions to prepare it for absorption, while maintaining an appropriate pressure gradient across the pyloric sphincter to regulate gastric emptying [1]. The two most prevalent gastric motility disorders are functional dyspepsia and gastroparesis, the cause and physiology of which are diverse [2]. Both have similar symptoms, including upper abdominal pain, nausea, and early satiety, and can be associated with delayed gastric emptying [2, 3] which can make it difficult to distinguish between them clinically [2]. Both are chronic disorders that generate significant health care costs [4], and in both cases new approaches are needed for minimally invasive, long-term, ambulatory monitoring to potentially improve diagnosis and management.

Gastric function studies that can identify delayed gastric emptying include scintigraphy, C13 breath tests, wireless motility capsule tests, and combinations thereof [5]. All these methods have benefits and drawbacks. Scintigraphy is currently the gold standard [3], which is limited by 4-hour stationary testing [6], and exhibits high intrapatient variability, largely dependent on body position [7]. C13 breath tests overcome the radiation issues of scintigraphy but can be influenced by other factors in the body and are limiting in terms of the meal that can be assessed [5]. Wireless motility capsule-based tests overcome the radiation limitations as well but do not offer gastric retention and therefore are better suited to assess colonic motility [8]. The method still has a potential issue of unintended retention, occasionally requiring endoscopic removal [5]. Gastric motility has been shown to be relevant to functional dyspepsia in clinical studies, with the gastric emptying rate exhibiting significant negative correlation with the severity of symptoms [9]. Functional MRI may be capable of providing information about antral contractile activity but is limited due to its short duration of assessment and high cost [10]. Antroduodenal manometry is another avenue for assessing foregut motor function and can be employed in long-term (>24 h) ambulatory tests [11] but is catheter-based (transnasal or transoral) and has demonstrated inconsistent performance in the larger antral lumen, and in many cases its results can be difficult to interpret. Noninvasive electrical-based measurements such as electrogastrography [12] (EGG) or bioimpedance [13] currently lack reliability, and so far the use of electricity in gastric motility studies has been considered to be not adequately validated for clinical use [14].

**Figure 1:**
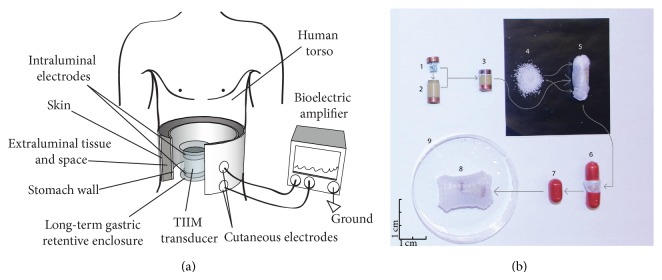
(a) The TIIM principle shown schematically: a small electrical signal is emitted from the TIIM transducer intraluminally from within the stomach after ingestion. The transducer is contained within an expandable pill that swells within the stomach, thus preventing it from being expelled through the pylorus for a prolonged amount of time (days). The attenuation dynamics of the electrical signal across the gastric and extraluminal tissue is measured from the skin via external cutaneous electrodes. The TIIM signals are measured using a standard multichannel bioelectric amplifier (electrogastrograph). After a predetermined amount of time (depending on the materials used), the long-term gastric-retentive enclosure disintegrates and the resulting smaller constituents of the pill individually exit the gastrointestinal tract via natural peristalsis. The TIIM components in the figure are deliberately zoomed in. (b) TIIM gastric-retentive pill design: (1) electronic oscillator circuit; (2) capsule body; (3) assembled capsule; (4) superabsorbent granules; (5) capsule and granules inside a liquid-permeable mesh; (6) dissolvable pill containing the meshed capsule; (7) TIIM gastric-retentive pill; (8) pill expanded in water; and (9) test dish. Horizontal and vertical 1 cm scales are depicted at the bottom left corner.

Transcutaneous intraluminal impedance measurement (TIIM) works by emitting a small high-frequency electrical signal from within the stomach, with parameters (50 kHz, 1.5 V peak-to-peak) chosen for their optimal transmission properties through smooth and abdominal muscles [15] and other extraluminal tissues, thus reaching the skin with potentially strong residual energy (Figures 1(a) and 1(b)). The steady, stable, and rectangular voltage signal is generated by a watch battery-supplied miniature electronic oscillator. Since the frequency and the originally generated amplitude of this broad-spectrum rectangular signal are known, it is possible to quantify its amplitude attenuation and related modulation by measuring the resultant signal at the surface of the skin using a plain electrogastrograph [16]. It is hypothesized that gastric contractions are amplitude-modulated on the high-frequency carrier, and TIIM reflects these contractions after appropriate amplitude demodulation. Two implementations of the gastric TIIM oscillator have been developed: a catheter-based system and an ingestible pill form that expands in the stomach and is retained for a long duration [16]. In both cases, pilot studies [16] have demonstrated that TIIM is capable of measuring gastric motility with comparable precision to invasively implanted force transducers on the stomach.

TIIM is a substantially different concept from standard electrogastrography (EGG), where cutaneous electrodes measure the intrinsic spontaneous electrical activity of the stomach. EGG has substantial drawbacks due to dynamic artifacts and lack of direct correlation with gastric muscular function, disease, or symptoms [14]. Conversely, the gastric-retentive TIIM measures the attenuation dynamics of a predetermined high-frequency signal originating within the hollow viscus of the stomach. Based on the TIIM signal's parameters, its measurement is completely independent of and unaffected by any intrinsic electrical activity, whether originating from the gut or elsewhere (e.g., the heart). Thus, for gastric applications, the major driver for the variations in TIIM signal attenuation is directly related to gastric contractions.

The aim of the present study was to evaluate the effectiveness of the minimally invasive, ingestible, gastric-retentive TIIM capsule for measuring gastric motility in comparison to a sham gastric-retentive, ingestible capsule, with both being referenced to force transducers attached to the serosa of the stomach in acute canine models [17]. This validation can be regarded as a necessary precursor to future long-term chronic ambulatory tests involving TIIM.

## 2. Methods

### 2.1. TIIM Capsule Design

For the present study, the TIIM miniature electronic oscillator was integrated into a gastric-retentive pill, which further lowers the already minimal impact of the catheter-based design [16]. Each custom-designed TIIM transducer contained a miniature, surface-mount, battery-supplied, 50 kHz/1.5 V electronic oscillator (Linear Technology, Milpitas, CA, USA). The length of the transducer was 18 mm with a diameter of 11 mm. The oscillating TIIM capsule had a battery life of 62 ± 3 hours. It was embedded in dry, biocompatible superabsorbent polymer granules contained in a nonwoven, permeable, 40-degree, 20 gsm polyvinyl alcohol (PVA) mesh (Qingdao TSKY Chemical Co., Ltd., Qingdao, China) inside a size AAA DB capsule (Capsugel, Morristown, NJ, USA). The polymer granules swelled to 30–40 times their dry size in gastric liquid. The parameters of the expandable capsule were chosen to have minimal impact on the stomach and to maintain ease of swallowing, while still being able to ensure gastric retention, even during interdigestive periods [8]. The permeable gastric-retentive capsule design has self-disintegration capabilities in 2-3 days, with no adverse mucosal impact or evacuation/obstruction issues, as previously described [18]. In addition, in this design we utilized temperature-controllable PVA mesh, which can be immediately disintegrated on demand by administering hot (>40°C) water.

### 2.2. Experimental Setup

#### 2.2.1. Animals and Animal Preparation

This study was approved by the Life and Environmental Sciences Animal Care Committee, University of Calgary, Calgary, Alberta, Canada.

Experiments were performed on eight mongrel dogs (6 F) with a mean weight of 23.8 kg ± 3.3 kg, four of which were administered an active TIIM capsule, while the rest were given a deactivated (battery-removed) capsule. After 24 h fasting and 12 h water deprivation, each animal ingested transorally a single capsule as described above (TIIM or sham) with 500 cc of room-temperature water. The pill swelled to its maximum size in the stomach within 15 minutes after ingestion to dimensions exceeding 1.5 cm in any direction and subsequently was unable to pass the pyloric sphincter even when subjected to propulsive peristalsis. The animals then underwent induction with an intravenous injection of thiopental (Thiotal 15 mg kg^−1^ IV, Vetoquinol Canada, Lavaltrie, QC, Canada) and were continuously maintained on inhalant isoflurane and oxygen (Halocarbon Laboratories, River Edge, New Jersey, USA) with a vaporizer setting of 1%–3% until the end of the experiment, so that gastric motility was minimally affected. The anesthesia was chosen because it did not influence gastric neurotransmitters and as such would not affect gastric contractions [19]. Individually, the animals were then positioned supinely, and their abdomens were shaved, cleaned, and sterilized with alcohol before performing laparotomy via a median incision vertically along the linea alba to gain access to the stomach.

**Figure 2:**
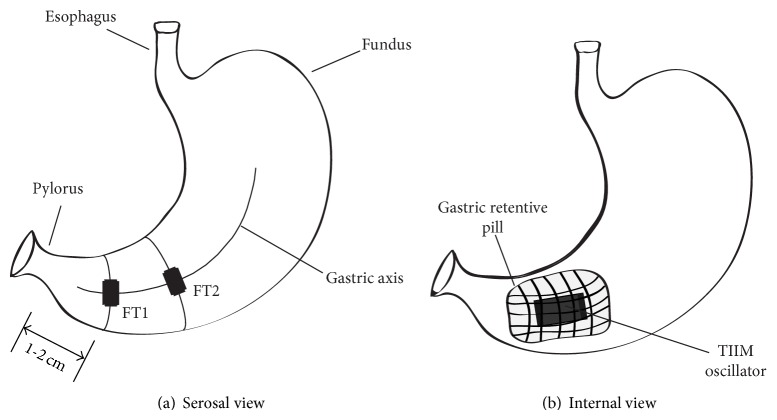
Position of the force transducers sutured to the serosa of the stomach (a) and the intraluminal position of the expanded gastric-retentive pill carrying the TIIM oscillator (b).

After the incision the location of the ingested pill in the stomach was verified endoscopically using an EPK-700 veterinary endoscope (Pentax, Tokyo, Japan), and the serosa of the stomach was measured using an oscilloscope (Tektronix, Beaverton, OR, USA) to confirm the presence of an activated or deactivated pill. After this verification, two 90W24 force transducers (RB Products, Stillwater, MN, USA), specifically designed for gastric motility monitoring, were surgically sutured to the serosal side of the antral stomach along the gastric axis [20]. The first force transducer was positioned 1-2 cm from the pylorus, and the second was affixed proximally 5-6 cm from the pylorus, along the gastric axis (Figure 2(a)). The mesenteric innervation and the blood supply of the stomach were carefully preserved. The intraluminal position of the gastric-retentive pill is shown in Figure 2(b).

The signals from the force transducers were amplified using a custom-designed multichannel bridge amplifier and digitized using a PCMCIA DAQ Card-AL-16XE-50 (National Instruments, Austin, TX, USA). The force transducer (FT) signals were monitored and analyzed with custom-designed signal processing and visualization software (GAS-6.2, Biomedical Instrumentation Laboratory, University of Calgary, Calgary, Alberta, Canada). Once the force transducers were in place, their functionality was verified mechanically by manual palpation of variable strength, and the offsets and gains were calibrated accordingly for maximal sensitivity. The intragastric position of the pill was then verified mechanically by palpating it to ensure that it had not been compromised during surgery.

Following the FT implantation the abdomen was closed, and after appropriate skin cleaning and preparation, three pediatric ECG electrodes (Conmed, Utica, NY, USA) were placed cutaneously over the stomach along the abdominal projection of the gastric axis, with a ground electrode positioned closer to the left hip of the animal [16]. The position of the electrodes was similar to the one associated with impedance epigastrography, since previous studies have suggested optimal electrode placement [21].

The cutaneous electrodes were connected to a custom-designed multichannel electrogastrograph (EGG, James Long Company, Caroga Lake, NY, USA), which measured the surface electrical activity relative to ground. The cut-off frequencies of the bandpass filter of the EGG amplifier were set to the commonly used 0.03–0.1 Hz following the hypothesis that gastric motility signals in the animals will not exceed 6 cycles per minute (cpm) [22] and will amplitude-modulate the intraluminal oscillator frequency of 50 kHz, with the latter acting only as their carrier. The 0.1 Hz low-pass filter would thus act as a demodulator for this transcutaneous signal transmission and would prevent higher frequency electrophysiological and mechanical processes (e.g., electrocardiographic activity and respiration) from interfering with the signal originating from within the stomach. The signals were then digitized using the same PCMCIA card DAQ Card-AL-16XE-50 (National Instruments, Austin, TX, USA) simultaneously with the FT signals and were subsequently monitored and stored for further analysis using the same custom software.

### 2.3. Experimental Procedure

Immediately after the experimental setup was completed [16], a baseline recording was performed with no pharmacological stimulant for 30 minutes. Following this recording, bolus neostigmine (0.04 mg kg^−1^, APP Pharmaceuticals, Schaumburg, IL) was administered intravenously as a smooth muscle stimulant to invoke contractions [20]. Thirty minutes of post-neostigmine recordings were subsequently obtained. The total recorded time from each animal was one hour, 1/2 hour basal state and 1/2 hour post-neostigmine state, with a one-minute time interval between them for the intravenous (IV) administration of the bolus neostigmine.

At the end of the experiments the animals were sacrificed by an IV injection of Euthanyl, 480 mg/4.5 kg (Bimeda-MTC Animal Health Inc., Cambridge, ON, Canada). Subsequent retrieval of the expanded pill was performed in order to verify its retention within the stomach and confirm the presence of the signal in the active TIIM pills or the lack thereof in the inactive sham pills using an oscilloscope. The postadministration volume of each gastric-retentive pill was measured to quantify expansion dimensions.

### 2.4. Signal Processing and Statistics

Since the cutaneous and the force transducer measurements were relativistic in nature, all measurements (2 cutaneous and 2 FT channels) were normalized with the maximal amplitude in 1-minute intervals becoming unity and the minimal set to zero. Thirty one-minute gastric motility indices [23] were calculated for each channel per test (basal or post-neostigmine) from the normalized measurements by calculating the total signal power of the normalized signal for each minute. The corresponding motility indices (TIIM versus FT) were comparatively assessed using the Pearson correlation coefficient (PCC) [24]. For each 30-sample correlation, a PCC of 0.349 or greater was considered to be statistically significant (*P* < .05) [24].

In addition, the frequency spectra of the signals were computed at 3.4-minute intervals with 64% overlap (2048-point fast Fourier transform, 10 Hz sampling frequency), and the frequencies of the dominant peaks per spectrum per modality (FT, TIIM, or sham-based EGG) were recorded and averaged utilizing the custom-designed software package GAS 6.2.

Furthermore, the dominant peaks of the frequency spectra for each measuring modality (basal and post-neostigmine) were subjected to a comprehensive statistical analysis using the paired Student's *t*-test [24] to evaluate the relationship between the frequency dynamics of TIIM, sham, and FT measurements.

## 3. Results

### 3.1. Gastric Retention

**Figure 3:**
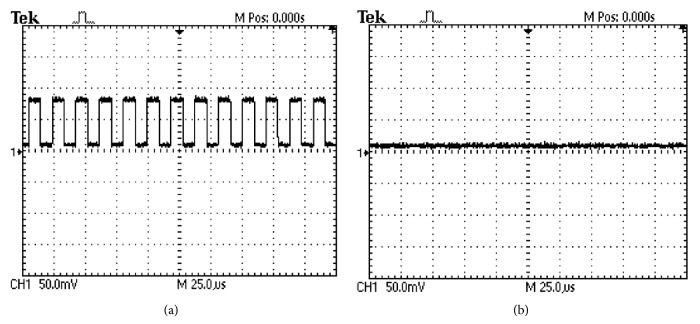
An oscilloscope reading from the gastric serosa prior to the force transducer implantation verified the presence of an activated TIIM pill (a). The sham pills did not have any signal (b).

Prior to the force transducer implantation, the electrical activity or lack thereof of the ingested pill was verified with an oscilloscope.  Figure 3(a) shows sample tracing of an active pill in the stomach measured from the serosa, while Figure 3(b) depicts sample oscilloscope tracing of an inactive sham pill. Final verification of the pill's activity at the conclusion of each experiment revealed that all TIIM pills had remained active for the entire duration of the test, and conversely, sham pills remained inactive as anticipated.

In each animal the expanded pill was retrieved from the stomach at the end of the experiment, with an average postretrieval volume of 12.1 ± 0.4 mL and dimensions exceeding 1.5 cm in all directions, indicating that even the neostigmine-invoked contractions had not been able to propel the expanded gastric-retentive capsule beyond the pyloric sphincter.

### 3.2. Motility Indices and Pearson Correlation Coefficients

**Figure 4:**
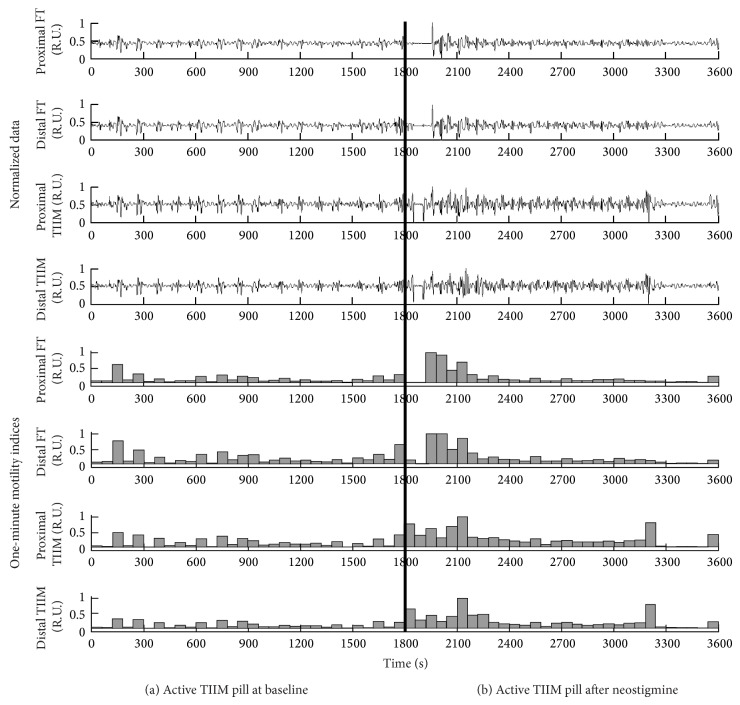
Combined plot of the raw signals and the 1-minute motility indices for an active pill in the baseline state (seconds 0–1800) and after the administration of neostigmine (seconds 1800–3600). The 1-minute duration for the administration of neostigmine is denoted by a thick vertical line.

**Figure 5:**
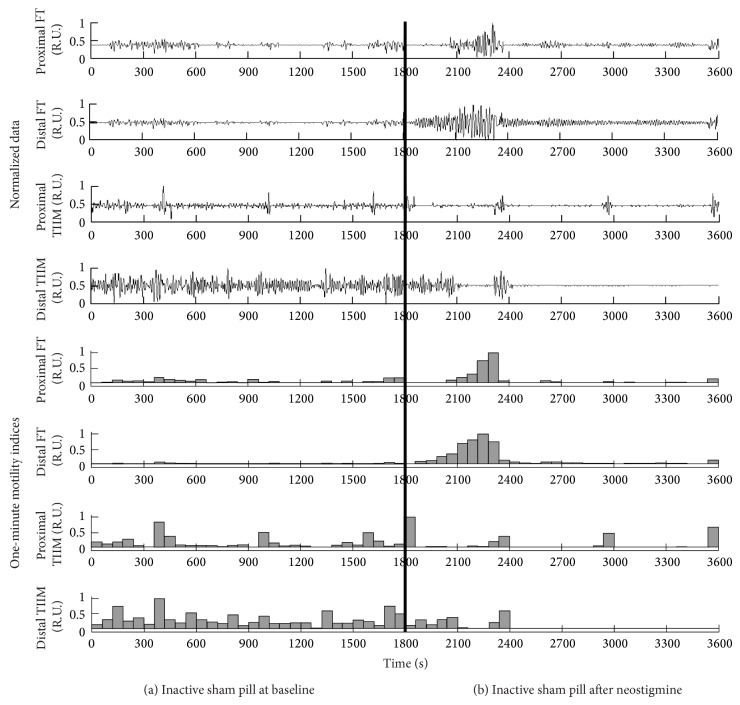
Combined plot of the raw signals and the 1-minute motility indices for an inactive pill in the baseline state (seconds 0–1800) and after the administration of neostigmine (seconds 1800–3600). The 1-minute duration for the administration of neostigmine is denoted by a thick vertical line.

A typical example of simultaneous FT and TIIM recordings for an activated pill, as well as their one-minute motility indices, is shown in Figure 4. The combined plots depict 30 minutes of basal activity, followed by 30 post-neostigmine minutes. During the baseline test there was varying evidence of spontaneous contractile activity. After the administration of neostigmine, the frequency, the regularity, and the amplitudes of the contractile activity increased significantly. In both cases (basal or induced contractions), there were statistically significant (*P* < 0.05) correlations between the TIIM gastric motility indices and the FT gastric motility indices. In the case of the deactivated sham pill (Figure 5) the results showed no statistically significant correlations between the respective motility indices.  Table 1 summarizes the averaged Pearson correlation coefficients (PCCs) of the one-minute gastric motility indices (GMIs) per state per capsule type.

Table 2 presents the averaged values of the dominant peaks of the frequency spectra (frequency range 0.03–0.1 Hz) of all recorded signals per modality (FT, TIIM, or sham-based EGG). The substantial dissociation between the averaged dominant frequencies of the FT and the electrical recordings in the sham capsule study is clearly evident, particularly during baseline when the contractile activity was sporadic and irregular. Comparative statistical evaluation of the frequency dynamics of the dominant spectral peaks (Table 3) revealed statistically significant relationship between TIIM and FT recordings (*P* < 0.05) [24]. Sham recordings (plain EGG) did not demonstrate such relationship to FT.

## 4. Discussion

The clinical utility of gastric electrical measurements (EGG or bioimpedance) for assessing gastric motility has been previously shown to be limited [12, 14]. The results of the present acute animal study validate TIIM against the gold standard of serosally implanted force transducers. TIIM can be regarded as intraluminally enhanced, invoked, nonphysiological electrogastrography, which in the present study appears to reflect gastric motility more accurately than the intrinsic EGG signal from the stomach (see Tables 2 and 3). Spontaneously generated intrinsic gastric electrical activity, manifesting itself cutaneously as the traditional EGG, best reflects the omnipresent, periodic gastric slow waves (known also as the electrical control activity or ECA [25], a result of the Na^+^-K^+^ ionic exchange at cellular level leading to cellular depolarization, which is a necessary but not sufficient condition for contractions to occur) [26]. Gastric contractions are associated with the calcium ionic influx at cellular level, electrical phenomenon known as electrical response activity (ERA), which occurs always synchronously with the ECA but not necessarily within each and every ECA cycle. ERA is a complex electrical phenomenon itself. Stronger contractions, reaching 0.5 gf, are associated with a larger plateau in the intracellular recordings of ERA. However, in the distal antrum the omnipresent ECA is occasionally accompanied not only by the ERA plateau but also by an additional high-frequency electrical signal superimposed over the latter. The appearance of this higher frequency electrical signal, known as “spikes,” has been associated with strong contractions exceeding 0.5 gf [27, 28]. Therefore, it has been suggested to separate the transmembrane dynamics of calcium ions into slow calcium transmembranic channels (the plateau or type I electrical response activity, ERA-I) and fast calcium channels (spikes or type II electrical response activity, ERA-II) [27]. Both ERA-I and ERA-II are not omnipresent, particularly in fasting. Moreover, extracellularly the plateau (ERA-I) and the spikes (ERA-II) get nonlinearly differentiated due to the properties of the cellular membranes resembling “leaky capacitors” [28]. Thus, extracellularly, both ERA-I and ERA-II, although associated with contractions, become of low average electrical power which is easily dissipated intraluminally in the body and cannot consistently significantly contribute to the power dynamics of the cutaneously recorded EGG to a point of being reliably detected. Therefore, in the complex interaction between the cutaneous representations of ECA, ERA-I and ERA-II, and the influence of numerous external factors including the body mass index of the patient, the skin impedance, and motion and motility artifacts, amplitude and power dynamics of EGG cannot consistently and reliably reflect amplitude and power dynamics of gastric contractions. In order to overcome this limitation of the otherwise attractive and noninvasive EGG technique, we decided to position within the lumen of the stomach a man-made oscillator of electrical power and frequency that would not be able to dissipate in the body in such inconsistent fashion as the intrinsic, spontaneously generated gastric electrical activity does. Thus, the TIIM-based electrical signal reaches the abdominal skin with easily detectable and reliably recordable parameters. The demodulation of this cutaneously recorded signal does not present any technological problem and can be achieved with an existing standard EGG amplifier. It is important to note that TIIM would not pick up or reflect ERA of any type, since the electrical dissipation of the latter would remain within the body. Instead, due to the movement of the gastric-retainable oscillator within the gastric lumen caused by contractions, the signal from this intraluminal oscillator would be amplitude-modulated in TIIM. So, TIIM is an electromechanical rather than an electrophysiological signal.

It is worth noting that although the aim of the present study was to further validate the TIIM technique, the sham pill study was fundamentally a direct comparison between gastric motility indices obtained via routine EGG and force transducers, since standard EGG bandpass filtering parameters (0.03–0.1 Hz) were used. In four animals no statistically significant correlation was observed in the motility indices between any of the channel pairs (FT-EGG) examined, further supporting our understanding that EGG cannot reliably monitor gastric motility due to its important and, in our opinion, unsurpassable limitation; the relatively low average electric-power of extracellular ERA-I and ERA-II preceding mechanically relevant gastric contractions dissipates intraluminally in an inconsistent and nonlinear fashion before reaching the abdominal skin surface and thus cannot be reliably recorded by traditional cutaneous EGG. With the electrically isolated, gastric-retentive pill-based design presented in this study, it is possible that environmental electrical phenomena such as capsule tumbling due to motion not associated with gastric contractions, static electricity, and external electromagnetic signals may affect the measurements in an ambulatory setting, particularly if the patient is mobile in an electromagnetically dynamic environment. In addition, in an ambulatory setting it is possible that abdominal muscle contractions and motion artifacts might also be registered. In such cases, adaptive filtering procedures [29] might help to eliminate the unwanted phenomena but at the price of increasing the complexity of the method and the number of recorded channels. Further considerations and potential advanced digital signal processing might be required postprandially, since solid or liquid meals could alter the base impedance and affect the efficacy of TIIM, calling for more powerful digital filtering and dynamic amplification of the TIIM signal. The limitations of the present study and indeed of this new TIIM technique require further evaluation to examine their impact on the clinical efficacy of this novel idea.

It should be noted that in the case of the inactive sham study small noise artifacts appear dramatic since there is no 50 kHz carrier signal as a reference. In fact, these noise artifacts were much lower (10–100 times) than the measurements from the active TIIM study. However, since the signals were normalized (the highest peaks were equated to unity and the lowest were equated to zero) before visualization and subsequent presentation, electrostatic noises affected the cutaneous recordings of the sham TIIM signals, and they appeared as enlarged white noise. Unfortunately, we cannot avoid normalization, because there is no exact way to determine the basal impedance the TIIM electrical oscillator faces, which can also be dynamically changing, particularly in chronic studies of longer duration. Regardless, it should be noted that if the sham TIIM tracings were displayed on the same scale as the active TIIM figure, they would appear as virtually flat lines. In future long-term (e.g., 24 h) chronic gastric motility studies utilizing TIIM, we do not perceive the normalization of the TIIM data to pose a significant problem, since the interdigestive motor complex and postprandial contractility would be clearly seen, particularly if a data logger marks the meal periods while recording the TIIM data.

The next steps for TIIM involve the development of a portable data-logger to enable ambulatory measurements in long-term chronic tests, which can examine the effectiveness of the method in more clinically relevant settings, in dynamic, noise-ridden environments and during meals. Further long-term chronic animal studies (>24 h) should be considered, potentially evaluating gastric motility and gastric emptying using TIIM, before moving to chronic human trials.

## 5. Conclusion 

Transcutaneous intraluminal impedance measurement (TIIM) is a minimally invasive gastric motility monitoring technique which has now been validated in a sham-comparative study. Acute canine models confirmed that TIIM was able to measure gastric motility with comparable precision to force transducers implanted invasively to the serosa of the stomach.

## Figures and Tables

**Table 1:** Averaged Pearson correlation coefficients (PCCs) of the one-minute gastric motility indices (GMIs) per state per capsule type [17].

Modality	State	PCCs proximal FT-proximal	*P* value	PCCs distal FT-distal	*P* value
		Cutaneous GMIs		Cutaneous GMIs	
TIIM capsule	Baseline	0.763 ± 0.2	<.01	0.674 ± 0.47	<.01
	After neostigmine	0.731 ± 0.12	<.01	0.734 ± 0.14	<.01

Sham capsule	Baseline	0.160 ± 0.03	>.1	0.071 ± 0.02	>.1
	After neostigmine	0.113 ± 0.09	>.1	0.051 ± 0.03	>.1

**Table 2:** Averaged cycles per minute (CPM) of the raw force transducer (FT) and the cutaneous recordings per state per capsule type.

Modality	State	Channel	CPM
TIIM capsule	Baseline	FT	2.38 ± 1.2
		TIIM	2.42 ± 1.27
	After neostigmine	FT	3.55 ± 0.94
		TIIM	3.58 ± 0.95

Sham capsule	Baseline	FT	2.65 ± 1.15
		EGG	3.94 ± 1.67
	After neostigmine	FT	3.84 ± 0.91
		EGG	4.12 ± 1.56

**Table 3:** Statistical comparison between the dominant frequency peaks of the FT and TIIM/EGG recordings using paired Student's *t*-test.

Modality	State	*P* value
TIIM capsule	Baseline (FT versus TIIM)	.048^*^
	After neostigmine (FT versus TIIM)	.049^*^

Sham capsule	Baseline (FT versus EGG)	.92
	After neostigmine (FT versus EGG)	.33

^*^(*P* < .05).
